# Bilateral patellar tendon reconstruction using LARS ligaments: case report and review of the literature

**DOI:** 10.1186/s12891-016-1161-1

**Published:** 2016-07-20

**Authors:** Adrian James Talia, Phong Tran

**Affiliations:** Orthopaedic Department, Western Hospital, Gordon Street, Footscray, Victoria 3011 Australia

**Keywords:** Patellar tendon, Rupture, LARS, Extensor mechanism, Reconstruction, Bilateral, Case report

## Abstract

**Background:**

Acute bilateral patellar tendon rupture is a rare occurrence, with only 1 case reported in the English literature of a young, fit athlete with no regular medications. To our knowledge this is the first such case reported using a LARS reconstruction.

**Case presentation:**

A 26-year-old otherwise well ex-olympic gymnast with bilateral acute on chronic patellar tendon rupture underwent reconstruction using LARS ligaments. At four years post-operatively he has maintained full range of motion and strength, without re-rupture or any evidence of synovitis.

**Conclusion:**

The use of LARS ligament for reconstruction of the patellar tendon is a viable and effective option for rupture. It avoids donor site morbidity associated with autograft. Reconstruction of both patellar tendons simultaneously in a young, elite-level athlete has not previously been reported in the English-language literature.

## Background

Bilateral patellar tendon rupture is an extremely rare occurrence, with only 50 cases present in the reported literature. There are few reported cases in the literature in a fit and otherwise well young patients [1–4]. Large tendinous rupture has commonly been reported in the context of systemic diseases such as diabetes mellitus, rheumatoid arthritis, lupus erythematosus, end stage renal disease and hyperparathyroidism [5–8]. Repeated or prolonged use of corticosteroids either systemically or locally may also predispose to tendon rupture. Many athletes are involved in repetitive sporting activities that place stresses on the extensor mechanism of the knee, however only a few suffer from “Jumper’s knee” and even fewer experience the end stage of this where the tendinous attachment of the patella is ruptured. Patellar tendon rupture tends to occur more commonly in those under the age of 40 years, whereas quadriceps tendon rupture tends to occur in those older than 40 years; there have been two reports of simultaneous patellar and contralateral quadriceps tendon ruptures [9–11].

Our aim herein is to present a case of bilateral patellar tendon rupture in a 26 year old gymnast which was repaired simultaneously with Ligament Augmentation Reconstruction System (LARS) ligaments. This is an extremely rare condition and to our knowledge this is the first case in the reported literature repaired using LARS ligaments.

## Case presentation

### Presentation

**Fig 1:**
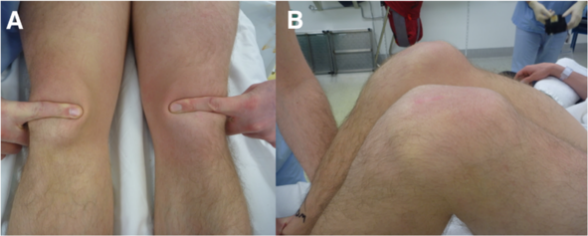
Photography of the pre-operative clinical examination, demonstrating a palpable defect superior to the tibial tuberosity (**a**) and bilateral patella alta (**b**)

A 26-year-old male ex-Olympic gymnast and gymnastics coach presented to our emergency department with bilateral knee pain and inability to straight leg raise secondary to jumping from the roof of his single story home, where his legs gave way after he landed on his feet. This is in the context of landing a jump vault and hearing a “popping sound” and sudden onset pain from his knees bilaterally 3 days prior. He had a background of chronic patellar tendonitis and a repair of his left patellar tendon for partial rupture 9 years previously. The patient was otherwise well and taking no regular medications. Physical examination revealed bilateral patella alta with a palpable defect superior to the tibial tuberosity; there were no significant joint effusions (Fig. 1).

### Investigations

**Fig 2:**
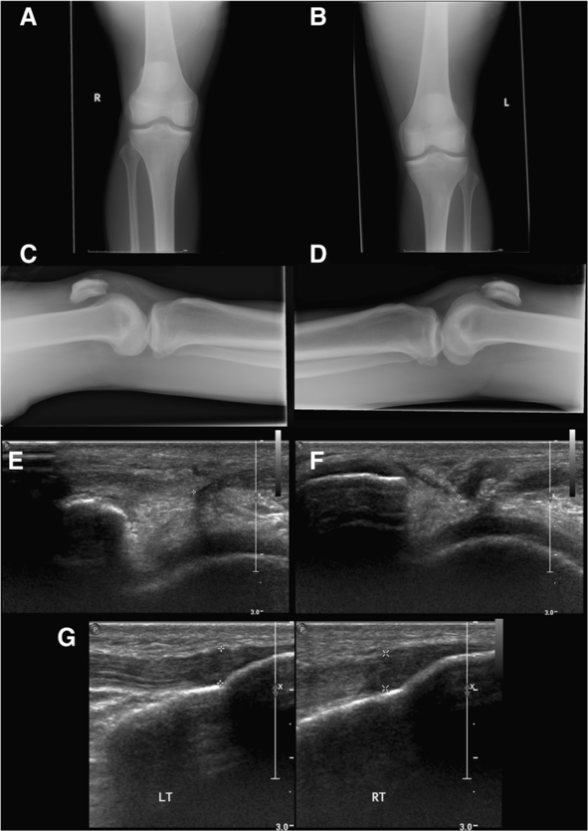
Pre-operative imaging demonstrating bilateral patellar tendon rupture: Xray imaging (**a**) Anteroposterior (AP) view of the right knee, (**b**) AP view of the Left knee. **c** Lateral view right knee and (**d**) lateral view left knee. These four images indicate bilateral patella alta. Ultrasound imaging is shown in: (**e**) Saggital view of the right patellar tendon demonstrating a 1.65 cm defect indicating rupture, (**f**) Saggital view of the left patellar tendon demonstrating a 1.49 cm retraction indicating rupture. **g** Bilateral views of the distal tibial insertions indicating that this was intact bilaterally

Xray demonstrated high riding patella bilaterally. The diagnosis was confirmed on ultrasound, with the right proximal insertional fibres ruptured and 1.5 cm retraction (Fig. 2). The right distal tibial insertion was intact. The left had a similar appearance with some calcified areas on the retracted fibres, which were believed to represent chronic calcification and/or avulsion fragments.

### Surgery

The patient was consented for a bilateral patellar tendon reconstruction rather than a primary repair due to the poor quality of the tendons. Autograft was not considered as the injury was bilateral and due to the volume of remaining tendon fibres. The aim being to return him to his elite gymnastics as soon as possible. Surgery was performed by the senior author (PT).

**Fig 3:**
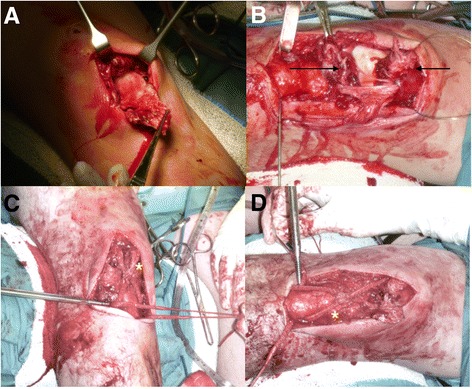
Intraoperative photographs from our bilateral patellar reconstruction: The exposed right (**a**) and left (**b**) (torn tendon ends indicated by arrows) patellar tendons demonstrating completely ruptured tendons. The right (**c**) and left (**d**) LARS ligaments (indicated by *) in situ during placement of the transverse tibial tunnels

Bilateral midline incisions were made over both knees, the operative findings confirmed the sonographic diagnosis. The reconstruction was performed using a ‘figure 8’ pattern through a transverse patellar tunnel with screw fixation to two tibial tunnels. The knee was placed at 90° of flexion prior to tensioning of the ligaments. The remnants of the patella tendon where sutured to the LARS ligaments using a braided, absorbable suture. The patella tendon was repaired using 2x corkscrew anchors (medial and lateral). The wound was closed in layers achieving meticulous haemostasis (Fig. 3).

**Fig 4:**
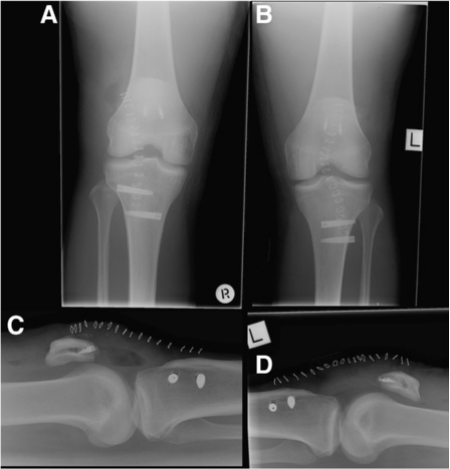
Post-operative Xrays demonstrating patellar and tibial tunnel placement. **a** AP view right knee, (**b**) AP view left knee, (**c**) Lateral view right knee and (**d**) lateral view left knee. These tunnels demonstrate the way in which the ‘figure 8’ reconstruction was performed

Post-operative Xrays indicated adequate screw and tibial tunnel placement (Fig. 4). The patient was allowed to weight bear as tolerated and placed in extension knee splints for 2 weeks to minimize the chance of wound dehiscence. Rehabilitation with physiotherapy started on day 1 focusing initially on passive range of motion (ROM) exercises then on extensor and quadriceps strength.

### Progress

At the two-week wound review post-operatively the patient still had some ongoing pain and swelling after bending the knees, however there were no signs of wound infection. The ROM of the right knee was 0-85° and the left 0-45°. At this appointment the patient was able to complete a straight leg raise bilaterally indicating an intact extensor mechanism. Two months post-operatively ROM was 0-125° bilaterally with no reported complications. At four months his ROM had improved to 0-130°.

**Fig 5:**
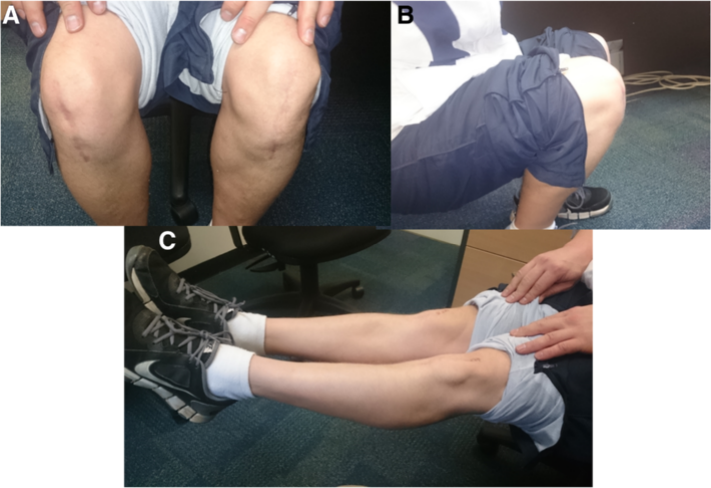
Physical examination at four years post-operatively demonstrates (**a**) bilateral well healed scars, (**b**) flexion to 130° bilaterally and (**c**) full extension bilaterally

At four years post-operatively, the patient is running a gymnastics club and actively coaching young gymnasts. He still experiences mild discomfort on descending stairs if carrying heavy loads but is otherwise painfree. He has not returned to training for competitive gymnastics. The patient claims he could return to some of the gymnastics skills but would struggle with vault and floor exercises secondary to lack of training. His Tegner Lysholm Knee score is 80, Modified Cincinatti rating score is 77 and Mohtadi-QOL Knee score is 58. Physical examination demonstrates well healed scars, no ligamentous instability or pain on palpation of the patellar tendon and bilateral ROM from 0-130° (Fig. 5).

## Discussion

Patellar tendon rupture is a debilitating injury. Despite spontaneous patellar tendon rupture *in an otherwise well patient* being a rare occurrence, there are some cases described in the literature [1–4]. To our knowledge, this is the only case reported in a young, fit athlete using a LARS synthetic ligament. If neglected or not correctly diagnosed, patellar tendon ruptures can become chronic and are technically difficult to repair [12, 13]. The management goal of this type of injury is to restore the mechanism that facilitates active knee extension; this is commonly achieved with end-to-end sutures or reconstruction to protect the injured tendon. Patients who undergo delayed repair are at risk of loss of full knee flexion and decreased quadriceps strength [13, 14]. Hence, swift treatment of fresh rupture is needed to prevent tendon retraction leading to quadriceps contractures and loss of extensor function. In chronic disease, or acute disease with poor tissue quality due to underlying disease or severity of injury, tendon grafts are often used to augment reconstruction. The use of hamstrings and tendoachilles autografts provide a viable option for successful reconstruction [2, 15, 16]. However the technique can be limited by adhesions, contractures and quadriceps atrophy post-operatively [17]. Thus in some cases artificial tendon grafts are preferred, avoiding the morbidity from donor site harvesting procedures. Extensor mechanism repair using carbon fibre had shown encouraging results in the late 1980s. More recently, extensor mechanism deficiencies using LARS reconstruction has been demonstrated to be effective in both patellar tendon ruptures [18] and after radical tumour resection [19].

The use of LARS ligament is well established in the case of other ligamentous reconstruction of the knee, particularly the anterior cruciate ligament (ACL), LARS has been shown to be advantageous for the early return to high-demand activities [20–22]. Advantages of the LARS ligament are as follows: (1) avoidance of donor site morbidity, (2) the ligament’s mechanical properties which allows early mobilization and quicker rehabilitation, (3) no evidence of graft rejection, (4) fibroblast ingrowth into the LARS matrix and (5) the possibility of repeating the reconstruction in case of failure [18, 23, 24]. There is no long-term data and a lack of quality randomized trials on LARS, in fact the effects of LARS on the natural healing process of a patellar tendon are unknown. Long-term data from other synthetic ligaments raise the possibility of long-term failure and iatrogenic osteoarthritis. LARS ligaments have previously been reported successfully in unilateral patellar tendon reconstruction in the elderly patient cohort [18]. Our report differs in that we report successful use with long term follow up in a high-demand young patient with bilateral rupture.

The use of a ‘figure 8’ patellar tendon repair has been previously described by Takazawa et al. The advantages of such a technique includes: no need for cerclage wiring, which obviates the need for a second procedure to remove foreign material. No need for formation of a large hole in the patella, increasing the risk of a fracture. Transmission of the load from the patella to the tibial tuberosity and allows for appropriate patellar tracking on knee flexion. Most importantly, early mobilization and rehabilitation is possible as this technique avoids post-operative casting [25]. Our report differs in that they describe a hamstrings autograft.

This report highlights some important points. Firstly bilateral patellar tendon rupture is uncommon but can be extremely debilitating. In our case the early recognition and surgical treatment allowed early rehabilitation, which provided a good surgical and functional outcome. Our case is in keeping with the evidence, with the bilateral LARS reconstruction allowing our patient to return to his day-to-day activities in the community. Although he is no longer able to perform multiple backflips with a floor routine or launch off the vault, he is able to complete a difficult routine on the trampoline.

## Conclusions

We report a case of the use of LARS ligaments for the reconstruction of an acute bilateral patellar tendon rupture in a high-demand, gymnast with successful long-term follow up as demonstrated by PROMs. The surgical technique presented herein in simple and reproducible and is the first description of its kind.

## Abbreviations

ACL, anterior cruciate ligament; LARS, ligament augmentation reconstruction system; PROMs, patient reported outcome measures; QOL, quality of life; ROM, range of motion
